# A Global Research Agenda for Adolescents Living With HIV

**DOI:** 10.1097/QAI.0000000000001744

**Published:** 2018-07-11

**Authors:** Alice Armstrong, Jason M. Nagata, Marissa Vicari, Cadi Irvine, Lucie Cluver, Annette H. Sohn, Jane Ferguson, Georgina Caswell, Lucy Wanjiku Njenga, Carlo Oliveras, David Ross, Thanyawee Puthanakit, Rachel Baggaley, Martina Penazzato

**Affiliations:** * Department of HIV and Global Hepatitis Programme, World Health Organization, Geneva, Switzerland;; † HIV Programmes and Advocacy, International AIDS Society, Geneva, Switzerland;; ‡ Division of Adolescent and Young Adult Medicine, Department of Pediatrics, University of California, San Francisco (UCSF), San Francisco, CA;; § Department of Social Policy and Intervention, Oxford University, Oxford, United Kingdom;; ‖ Department of Psychiatry and Mental Health, University of Cape Town, Cape Town, South Africa;; ¶ TREAT Asia/amfAR—The Foundation for AIDS Research, Bangkok, Thailand;; # Healthy Adolescents & Young Adults Research Unit, Africa Health Research Institute, Mtubatuba, South Africa;; ** International HIV/AIDS Alliance, Cape Town, South Africa;; †† Positive Young Women Voices, Kenya;; ‡‡ Adolescent Treatment Coalition, Geneva, Switzerland;; §§ Department of Maternal, Newborn, Child and Adolescent Health, World Health Organization, Geneva, Switzerland; and; ‖‖ Center of Excellence Pediatric Infectious Diseases and Vaccines, Chulalongkorn University, Bangkok, Thailand.

**Keywords:** adolescents, HIV, research agenda, HIV testing, HIV treatment, HIV service delivery

## Abstract

**Background:**

Despite growing interest in undertaking research in adolescent HIV, the current pace of interventional research in particular remains very low compared with the needs of adolescents living with HIV (ALHIV). More robust evidence is needed to inform innovative and targeted interventions that bridge research gaps, inform policy, and improve outcomes for adolescents. A global research prioritization exercise was undertaken by WHO and CIPHER to focus efforts on priority research in the context of diminishing resources.

**Methods:**

The Child Health and Nutrition Research Initiative (CHNRI) methodology was adapted and used. Outcomes were reviewed by an expert group and 5 priority themes identified for testing, treatment, and service delivery, accounting for existing policies, published literature, and ongoing research.

**Results:**

A total of 986 research questions were submitted by 323 individuals from 67 countries. For HIV testing, priority themes included strategies and interventions to improve access, uptake, and linkage to care, and self-testing, particularly for key populations. For treatment, priorities included strategies to monitor and improve adherence, novel drug delivery systems, preventions and management of coinfections, optimal drug sequencing, and short- and long-term outcomes. For service delivery, priorities included service delivery models across the cascade, strategies to improve retention in care and sexual and reproductive health, support for pregnant ALHIV, and the provision of psychosocial support.

**Conclusions:**

This prioritized research agenda assists in focusing future research in ALHIV and will help to fill critical knowledge gaps. Key stakeholders, donors, program managers, and researchers should all support these priority questions and themes to collaboratively drive the adolescent HIV research agenda forward.

## Abstract 2

Supplemental Digital Content is Available in the Text.

## INTRODUCTION

Adolescents are now recognized as a distinct population with different health requirements from children and adults. Their new prominence in the health response is evident in current global health and HIV agendas, including the *United Nations Global Strategy for Women's, Children's, and Adolescents' Health*^1^ and *Start Free, Stay Free and AIDS Free*^2^ super-fast-track agenda. Such initiatives are advancing the goal to end AIDS by 2030, ensuring universal access to services and targeted interventions for adolescents.

Worldwide, an estimated 2,100,000 [1,400,000–2,700,000] people aged 10–19 years were living with HIV in 2016, 80% of whom were residing in Sub-Saharan Africa.^3^ With high numbers of estimated new infections among older adolescents (15–19 year olds) and many of the 920,000 children receiving antiretroviral therapy (ART) surviving into adolescence,^4^ HIV programs have increasingly recognized adolescents as a critical age group. Nonetheless, adolescents continue to be underserved by current services across the HIV cascade. Adolescents have significantly inferior access to and coverage of ART, higher rates of loss to follow-up (LTFU), poor adherence, and increased needs for psychosocial support and sexual reproductive health (SRH) services.^5,6^

Despite growing interest in undertaking research in adolescent HIV, the current pace of interventional research in particular remains very low compared with the needs of adolescents living with HIV (ALHIV).^7–9^ Considerable effort is still required to understand what works best for this population. More robust evidence is needed to inform innovative and targeted interventions that inform adolescent HIV policy. This will improve outcomes for adolescents and help reach global targets for an AIDS-free generation by 2030.^2^ Due to limited funding for HIV, there is a need to optimize available resources by focusing research efforts on priority areas with the greatest impact for this population.

The World Health Organization (WHO) and the Collaborative Initiative for Paediatric HIV Education and Research (CIPHER) of the International AIDS Society (IAS) have undertaken a global research prioritization process on ALHIV with broad engagement of global stakeholders. This article aims to describe the outcomes from this process and to highlight considerations for its implementation.

## METHODS

The CHNRI methodology, a well-established approach for setting health research priorities, was adapted and used for this process and is described in detail by Irvine et al^10^

In brief, 5 phases were performed to set research priorities for ALHIV as follows:

- A diverse, multidisciplinary expert working group was established to define the scope of the exercise. The process covered testing, treatment, and service delivery. HIV prevention was not included.
- An online survey to collect priority research questions was conducted using snowballing and targeted dissemination to reach a broad range of stakeholders. Respondents were asked to tag their questions within the relevant research area (testing, treatment, or service delivery) and the research domain (descriptive, discovery, development, and delivery—Table 1).
- The data were cleaned and sorted in Excel, and thematic coding and analysis of questions submitted was undertaken by A.A. and C.I. with the additional technical support from M.P. and M.V. A condensed list of research questions was formed to best reflect the breadth and detail of those submitted by respondents.
- In a second survey, respondents of survey one were invited to score the collated lists of research questions against predefined criteria (ie, answerability, impact, implementation, and equity). For each research question, participants could score the criterion as either yes (score = 100), possibly (score = 50), no (score = 0), or leave this blank if they did not feel sufficiently informed to judge. Rankings were based on the total Research Priority Score (RPS), which was computed as the mean of the scores for the different criteria, weighed according to published guidelines from CHNRI stakeholders and adjusted to a 100-point scale. In addition, Average Expert Agreement (AEA) scores were calculated, which represent the average proportion of scorers that agreed on responses for each of the 4 criteria.
- The outcome of the CHNRI process was then reviewed by an adolescent HIV expert resource group charged to identify the 5 priority themes emerging from the top 10 ranked research questions in each topic area (ie, testing, treatment, and service delivery). This was considered in the context of existing policies, systematic reviews, recently published research, and planned or ongoing research.

**TABLE 1.**
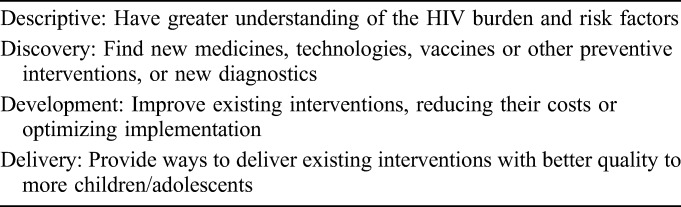
Research Question Domain Type

## RESULTS

A total of 986 research questions were submitted by 323 individuals from 67 countries across all WHO regions.^8^ After thematic content analysis, the final collated lists included 61 questions. The top 5 priority themes are described in Table 2. The final 5 themes identified in each area by the end of the exercise address the following. For HIV testing, priority themes included strategies and interventions to improve access, uptake, and linkage to care, and self-testing, particularly for key populations. For treatment, priorities included strategies to monitor and improve adherence, novel drug delivery systems, prevention and management of coinfections, optimal drug sequencing, and short- and long-term outcomes. For service delivery, priorities included service delivery models across the cascade, strategies to improve retention in care and sexual and reproductive health, support for pregnant ALHIV, and the provision of psychosocial support.

**TABLE 2.**
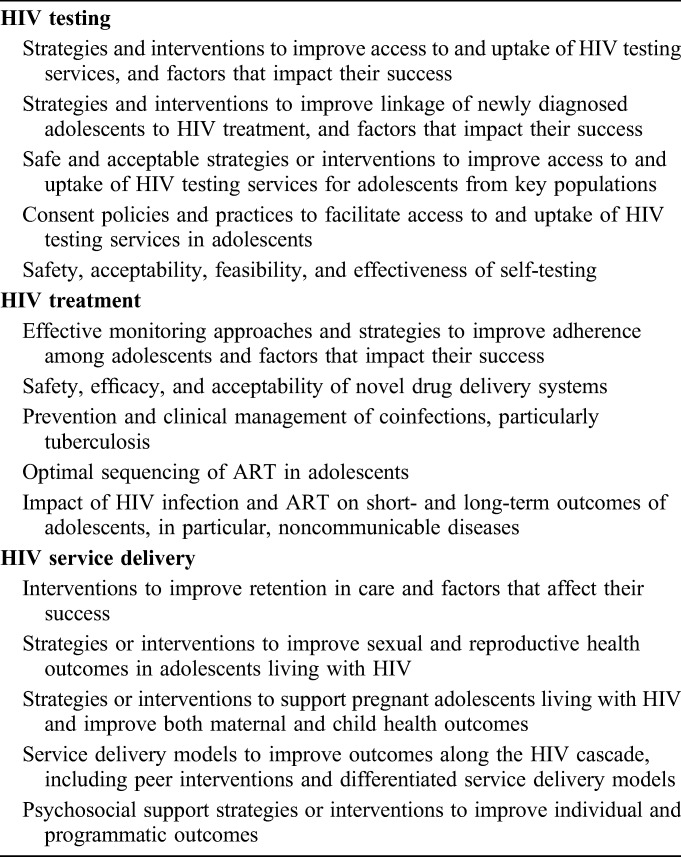
Top Five Priority Themes for Adolescent HIV Testing, Treatment, and Service Delivery

The full list of questions per topic area is provided in Table 1, Supplemental Digital Content, http://links.lww.com/QAI/B168. A total of 12 research questions on HIV testing were scored by respondents (n = 66). Among these, development (n = 4), delivery (n = 3), and descriptive (n = 3) type research questions were ranked similarly, with no discovery questions making the top 10. Respondents (n = 75) scored 17 research questions on treatment. Development (n = 6) was ranked more highly over descriptive (n = 2) and discovery (n = 2), with no delivery questions featured in the top 10. For service delivery, a total of 32 research questions were scored by respondents (n = 107). Development (n = 7) and delivery (n = 3) type research questions were ranked among the top 10, and no discovery or descriptive questions were included. For the top 10 ranked questions per research area, the overall mean RPS was 83–87 and the mean AEA was 69–75, indicating collective agreement around the top 10 high priority questions. Generally, the questions with the greatest level of overall agreement also achieved higher overall RPSs.

## DISCUSSION

This is the first broad research prioritization exercise conducted on ALHIV using a modified version of the CHNRI priority setting method. We have identified priority questions and themes for research in adolescent HIV testing, treatment, and service delivery. Wide engagement of diverse stakeholders has identified research questions that will be invaluable in guiding the future research agenda in adolescent HIV testing, treatment, and service delivery. A novel addition to the CHNRI method was the identification of 5 priority themes per research area considered in the context of existing policies, systematic reviews, recently published research, and ongoing research.

### HIV Testing

HIV infections during adolescence continue to occur at a high rate, with older adolescents, girls, and those from key populations at greatest risk.^11^ Barriers preventing adolescents from accessing HIV testing services—in particular, age of consent policies—were identified as a priority theme to investigate for improving HIV testing uptake. Although progress has been made in a number of Sub-Saharan African countries, according to a recent global review, most countries' age of consent for HIV testing remains between 16 and 18 years.^12^ For adolescents from key populations, these laws and policies are even greater barriers to accessing testing services because of the threat of possible prosecution of certain behaviors in some settings (eg, male–male sex, drug use, selling of sex).^13^

Evidence of effective interventions to increase uptake of testing and linkage to care for this age group is limited and emerged as key themes to investigate. A recent review by Bumgarner et al^8^ only included 5 interventional studies for those aged 10–24 years. Similarly, a review looking at HIV testing approaches for both children and adolescents found that studies used approaches developed for adults and did not consider the developmental and age-specific needs of the target populations; therefore, optimal testing approaches remain a priority theme to address.^14^ Although provider-initiated testing and counseling has been identified as a successful approach, adolescents may perceive themselves as healthy and therefore encounter fewer providers and clinic visits.^14^ HIV self-testing is a promising approach that emerged as a priority theme and is recommended by WHO.^15^ Three recently published studies indicated that uptake, acceptability, and fidelity of HIV self-testing was high in these age groups; however, they did not completely provide uptake or yield in substantial numbers of adolescents.^16–18^

### Treatment

Strategies to sustain high levels of adherence to ART emerged as a top priority theme to investigate for adolescent treatment. Reviews on adherence and virological suppression among adolescents highlighted varied yet inferior outcomes with unique influencing factors.^5,19,20^ Participants in a global adolescent community consultation on HIV treatment highlighted the complexities faced by adolescents taking ART daily.^21^ Similarly, a situational analysis across Sub-Saharan African facilities identified nonadherence as the key challenge in providing services for adolescents.^22^ Need for interventions to support ART adherence is clear; however, evidence to support specific approaches is limited and of low quality. MacPherson et al^7^, over a 13-year period, identified 5 evaluated service delivery interventions to improve adolescents' adherence. Most of the studies were conducted in high-resource settings, had small sample sizes, and lack of comparison groups, leading the authors to conclude calling for rigorous evaluation of existing and innovative interventions to support adherence.

Monitoring adherence is an essential step to identifying difficulties before treatment failure. However, with the unreliable nature of self-reporting or pill counting and limited access to routine viral load monitoring, objective adherence monitoring remains difficult for programs.^23,24^ This requires improved understanding of effective monitoring tools. In addition, drug-related strategies are imperative in optimizing treatment options and supporting lifelong adherence. Regimen simplification, harmonization with adult regimens, and identification of optimal sequencing of antiretrovirals are key to preserve future treatment options.^25^ The recent introduction of dolutegravir-based regimens in a fixed-dose combination promises to finally offer a well-tolerated, single-tablet, once-daily regimen with a high barrier to resistance. However, potential alternative strategies, such as structured treatment interruptions, weekends-off, or simplification to once-daily for twice-daily regimens, should be explored to support adherence in this population.^26^ In addition, the introduction and development of new drugs and novel delivery systems such as long-acting agents, which are currently under development for adults, also hold promise for adolescents.^27^

Evidence on coinfections among adolescents is insufficient and, management of coinfection, in particular for those with advanced disease, is a priority theme to investigate. The limited evidence on tuberculosis among ALHIV indicates nonadherence and poor outcomes, especially for those with multidrug-resistant tuberculosis.^28^ In addition, HIV has been associated with long-term complications for vertically infected adolescents, which include but are not limited to cognitive impairment^29^; poor lung function and chronic lung disease^30–32^; delayed pubertal onset and growth failure^33^; cardiovascular disease and metabolic complications^34,35^; and poor bone health.^36^ These findings underscore the need for further research on the long-term impact of HIV and ART during this critical time of development.

### HIV Service Delivery

Adolescents are at high risk of LTFU from HIV services, causing them to miss out on life-saving treatment, care, and support. An increasing number of cohort and programmatic analyses indicate that adolescents, particularly those aged 15–19 years and young adults (20–24 years), have higher LTFU rates, both before and after ART initiation, when compared with other age groups.^6,37^ Of concern, lower service uptake, LTFU, and higher mother-to-child transmission rates have been reported among HIV-infected pregnant and breastfeeding adolescents compared with HIV-infected adult mothers.^38^ Interventions to support adolescents' retention in care was identified as a research priority. MacPherson et al's^7^ review of service interventions across the cascade identified only 2 studies for linkage to care and retention. Their results suggested that improved accessibility to facilities, availability of youth-friendly services, multidisciplinary adolescent HIV clinics, and peer interventions warrant further investigation. Currently available evidence for differentiated HIV service delivery (DSD) for adult Community Adherence Groups indicates that younger participants, 16–24 years, were at higher risk of LTFU from the adherence group and the facility.^39^ With a growing programmatic shift toward DSD, evidence on how to implement targeted DSD for adolescents, especially with peer and community interventions, is needed.^40^

Physical, social, and psychoemotional changes experienced during adolescence are further compounded for ALHIV who must deal not only with managing a chronic condition, but also with the impact of a highly stigmatized illness on their sexual health, relationships, and emotional well-being.^20^ A number of studies have highlighted the multifaceted psychosocial stressors experienced by this population as well as the prevalence of mental health challenges.^9,41^ In addition, ALHIV SRH needs are not being met because specific services and information are insufficient and often inaccessible.^42,43^ Despite the increased awareness of the requirements for support, few studies have tested and compared different interventions. New and optimized strategies to address ALHIV psychosocial support, mental health, and SRH needs are urgently needed both at individual and program level.^9,42^

### Considerations for Implementing the Research Agenda

For the research agenda to reach its highest impact, researchers, funders, implementers, communities, and adolescents will need to share collective responsibility for its implementation. Table 3 provides suggested actions to support the adoption of the agenda.

**TABLE 3.**
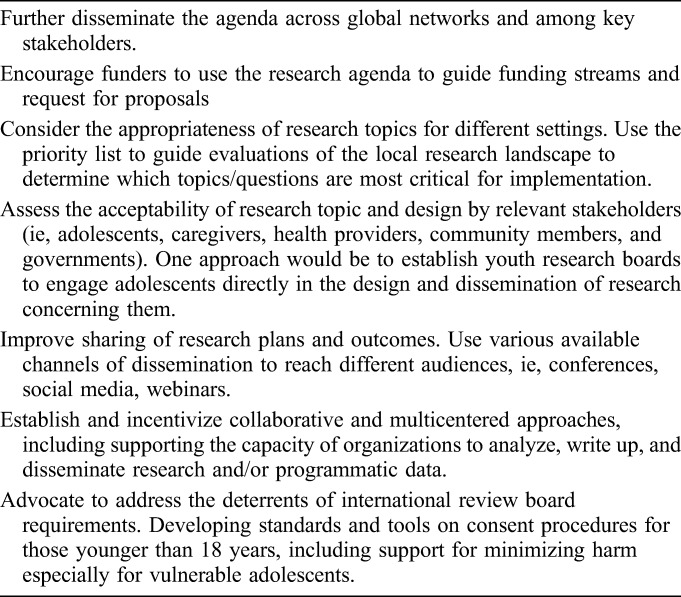
Suggested Stakeholder Actions to Support Implementation of the Prioritized Research Agenda

### Limitations

Many of the limitations within this research prioritization exercise are intrinsic to the CHNRI methodology. The methodological limitations are described in greater detail accompanying methods from the study by Irvine et al.^10^ The identification of the priority themes may have led to the omission of other research questions. The themes were developed in consideration of the current research context for ALHIV. The full list of prioritized research questions is available as a separate supplementary table. The strength of the processes lies in the contribution of a large number of research questions from a broad cross-section of geographically diverse and multidisciplinary stakeholders.

## CONCLUSION

This is a critical time for research on ALHIV. Adolescent health is increasingly at the forefront of the global agenda.^1^ Given the need for evidence-based policies and programs to improve adolescent HIV outcomes, priority themes and questions for research in adolescent HIV testing, treatment, and service delivery have been identified using a transparent process involving experts from diverse disciplines, types of institutions, and countries. The prioritized themes identified from the CHNRI process are largely consistent with current evidence gaps highlighted by the literature reviewed for the process. The implementation of the agenda will help to fill critical knowledge gaps and will be essential for reaching global targets for an AIDS-free generation by 2030. Key stakeholders, donors, program managers, and researchers should all support these priority questions and themes to collaboratively drive the research agenda for ALHIV forward.
